# The Effort-Reward Model and Its Effect on Burnout Among Nurses in Ecuador

**DOI:** 10.3389/fpsyg.2021.760570

**Published:** 2021-11-23

**Authors:** Luis E. Alvarado, Francisco D. Bretones, Juan A. Rodríguez

**Affiliations:** ^1^School of Psychology, University of Guayaquil, Guayaquil, Ecuador; ^2^School of Labor Relations and Human Resources, University of Granada, Granada, Spain; ^3^Program Prometeo-Senescyt, Merida, Ecuador; ^4^School of Psychology, University of Los Andes, Mérida, Venezuela

**Keywords:** burnout, effort-reward imbalance, emotional exhaustion, rewards, seniority, work stress

## Abstract

Burnout has harmful consequences for individuals and organizations. The study of its antecedents can help us to manage and prevent it. This research aims to explore the role of the effort-reward imbalance (ERI) model as well as the mediation of the working experience in the burnout processes. For this purpose, we have conducted a study in 629 employees from two hospitals in the city of Guayaquil (Ecuador). For this study, the Spanish version of the Maslach Burnout Inventory was applied, as well as the ERI Questionnaire, along with other socio-demographical and occupational variables. A statistical analysis was performed with the obtained data, using structural equation models (SEMs). Results showed that employee effort has a stronger and statistically significant direct effect on emotional burnout, whereas the perception of the obtained reward also had this effect but indirectly in a negative sense, with job experience as a mediating variable.

## Introduction

The study of burnout in jobs in direct contact with the public is well-known in the scientific literature (Van Mol et al., 2015; Giorgi et al., 2017; Salvagioni et al., 2017). In addition, it is known that burnout occurs as a response to chronic emotional and interpersonal stressors of the working activity. It consists of three components (Maslach and Jackson, 1981; Maslach et al., 2001): tiredness or emotional exhaustion (which represents the basic dimension referred to the feeling of lacking emotional and physical resources to confront the perceived stressors), depersonalization (which represents the interpersonal dimension and expresses excessive detachment, cynicism and insensitivity toward users, and other aspects of work) and diminished personal accomplishment (which represents the self-assessment dimension referred to the feelings of incompetence and lack of personal fulfilment and productivity at work).

Thus, the causes of burnout must be sought not only in objective conditions (Garcia et al., 2015) but mainly, in the individual’s interaction with those conditions, mediating by other psychosocial variables, such as personality, emotional regulations, as well as the perceptions, evaluations, and expectations of people (Salami and Ajitoni, 2016; Bang and Reio, 2017; Hwang et al., 2019; Ortega-Jiménez et al., 2021).

Several authors agree on the adverse job characteristics that are associated with burnout processes, especially in nursing employees (Dall’Ora et al., 2020; Almodibeg and Smith, 2021; Boateng et al., 2021). In addition, the negative consequences that this exhaustion process has both on organizations, affecting the quality of the service they provide (Leon-Perez et al., 2016), and on their lives, developing mental (Ding et al., 2014) and psychosomatic disorders (Guerrero-Barona et al., 2020).

Nevertheless, the influence of other work-related and organizational variables in burnout processes has been less studied. Some studies showed that the work overload can cause in the employees a perception of inability to satisfy the demands required by the staff or the organization (Peiró et al., 2001; Portoghese et al., 2014).

From this perspective, the so-called effort-reward imbalance (ERI) theory, proposed by Siegrist (1996), establishes that work stress would be generated from the lack of correspondence between a high level of effort that work requires and low rewards, both material and psychosocial, that the employee perceives. It is clear that the overload and work rhythms, the lack of support or the absence of fair rewards are some of the new psychosocial risks at work that are going to affect the well-being of workers (Leka et al., 2017; Alvarado and Bretones, 2018). This lack of reciprocity between the high cost and the low perceived benefit will cause strong negative consequences for the organizational results as well as personal consequences for the workers (Devonish, 2018). Among the organizational consequences, we can mention the intention to quit (Leineweber et al., 2021), perception of injustice at work (Topa et al., 2016; Juvani et al., 2018) or burnout processes (Oren and Littman-Ovadia, 2013; Backhaus et al., 2018; Jachens et al., 2019).

Among the personal consequences of this imbalance, we can mention: long-term implications for employee health (Siegrist and Li, 2016), such as development of coronary diseases (Gilbert-Ouimet et al., 2014; Dragano et al., 2017), higher levels of anxiety (Eddy et al., 2018) and depression and mental illnesses in general (Rugulies et al., 2017; Koutsimani et al., 2019; Diekmann et al., 2020), among others.

Also, other authors have observed that those workers who experienced ERI presented high levels of emotional exhaustion (Bakker et al., 2000; Basińska and Wilczek-Rużyczka, 2013; Tian et al., 2021).

Therefore, we propose the following hypothesis in this study:

**H1:** There is a positive relation between high levels of work effort and burnout.

**H2:** There is a negative relation between high levels of work reward and burnout.

However, in this relationship between ERI and burnout, we have to take into account the existence of other mediating or moderating organizational variables. Some of these studied variables had an occupational character, such as home-work interference (Gorgievski et al., 2019) or working shifts (Xie et al., 2011).

An example of this variable could be the job experience in the occupation. In any case, the results obtained in several studies about the relations between seniority and burnout are contradictory. Thus, some authors found that the number of years of stressing work, especially in emotional works, increased the experienced stress and in consequence, the emotional exhaustion (Makara-Studzińska et al., 2020). Nevertheless, other authors pointed out a reverse relation in such way that the younger workers and therefore, with less experience, that start their professional careers, have less realistic believes that make them more exposed to occupational exhaustion.

Therefore, we propose the following hypothesis in this study:

**H3:** The years of experience will have a significative mediating function in the relation between the ERI model and burnout. From this hypothesis, two sub hypotheses would arise:

**H3a:** The years of experience will have a mediating function by increasing in a significative and positive way the relation between effort and burnout.

**H3b:** The years of experience will have a mediating function by increasing in a significative and negative way the relation between reward and burnout.

## Materials and Methods

### Participants

In order to achieve our objective, we carried out a study in nurses from two hospitals of the city of Guayaquil (Ecuador). The participants were selected by non-probabilistic and accidental sampling method, being the inclusion criterion to be working as a nurse, with more than 1 year of job experience in the category and who was working on the day of data collection.

Finally, a total of 629 surveys were conducted. However, 70 questionnaires (11.1%) were subsequently rejected in the data analysis for different reasons (incomplete data, choice of several answers on the same item, and non-response). The final study sample consisted of 559 nurses from two hospitals (45.9 and 54.1%, respectively).

The age of participants ranged between 19 and 70 years old, with an average age of 42.06 years old and a standard deviation (SD) of 12.41. On the other hand, the seniority of employees participating in the study ranged between 1 and 42 years, being the average work experience 12.37 years old and a SD of 11.18. Distribution by gender shows predominance of the female sex in the study sample since 84.2% are women, compared to 15.8% who are men. Regarding the duration of the contractual relationship, the majority of the participants had a permanent contract with the organization (81%) compared to 19% who had a temporary contract.

### Variables and Instruments

For this research, the following measurement instruments were used:

In order to measure the burnout variable, we used the Spanish validation by Olivares-Faúndez et al. (2014) of the Maslach Burnout Inventory – Human Service Survey (MBI-HSS) (Maslach and Jackson, 1981). This 25-item scale is divided into three subscales (emotional exhaustion, personal accomplishment, and depersonalization) and measures how often people feel emotionally overloaded or exhausted because of their work. The inventory uses a scale ranging from 1 (never) to 7 (every day). The reliability index of this instrument, measured through the Cronbach’s alpha coefficient, was 0.81 in our study. This questionnaire is one of the most widely used in studies on this syndrome (Wheeler et al., 2011; Cañadas-de la Fuente et al., 2014).

We also applied the version adapted to Spanish by Juárez et al. (2015) of the ERI Questionnaire (Siegrist et al., 2004). In this study, we have only taken into account two of its dimensions: effort, which has 6 questions and records information about the employee’s assessment of his/her work situation in relation to the effort made at work; and reward, which has 11 questions that measure the professional reward. These 17 questions were formulated on a Likert-type scale with a standardized measurement format ranging from 1 (it does not affect me at all) to 4 (it affects me a lot). The Cronbach’s alpha index obtained in this study was 0.84, whereas each dimension had an alpha coefficient of 0.79 (Effort) and 0.75 (Reward). The ERI Questionnaire is one of the most widely used (Hanson et al., 2000; Rantanen et al., 2013; Siegrist et al., 2014).

Finally, we considered the following socio-demographical variables: gender, age, and job experience. The mediating factor job experience was measured in terms of the number of years held in the profession as nurse.

### Procedure

Concerning the procedure, we initially requested authorization from the Ethics Committee of the hospitals (#CE-0718), arranging a particular place and date for the collection of information.

On the date of the information collection, participants were informed about the purpose of the research, trying not to transmit information that could prompt responses to the questions included in the questionnaires.

Every potential participant agreed to collaborate. Instruments were administered in the above-mentioned order by self-application.

### Data Analysis

The collected data were processed and analyzed using SPSS^©^ 20.0 (IBM Company, Chicago, IL, United States) and AMOS 20.0 (SPSS Inc., Chicago, IL, United States). The statistical program SPSS version 20 (IBM, Chicago, IL, United States) was applied to calculate the descriptive analysis (frequencies, mean scores, and SDs). The relationships between variables were analyzed through correlation analyses and regression model using structural equation models (SEMs) and standardized fit indexes. Conventional levels of acceptable model fit [GFI, AGFI, NFI, and CFI, values over 0.85; root mean square error approximation (RMSEA) values >0.05] were taken into consideration (Browne and Cudeck, 1993; Chen et al., 2008).

The fit of the SEMs is determined by the fit indices that show the degree of concordance between the data predicted by the model and the observed data. The fit indices contribute to measure if the model fits well enough to provide a useful approximation to reality and a reasonable explanation of the data trends.

In addition, given that the value of χ^2^ is affected by the sample size (Barrett, 2007), we used the fit index CMIN/DF, which is less sensible and on the condition that the value was <3 (Hair et al., 2009).

In general, the SEMs allow us to test all the supposed direct and indirect effects among several variables simultaneously. These models are a superior alternative to others for proving mediations such as simple regression analysis, especially in cases where there are multiple indicators for each construct (Iacobucci, 2008).

### Declaration

This study was conducted in accordance with the Helsinki Declaration and the Good Practice Guide. The protocol was approved by the management of the participating hospitals. The study instructions were given to participants in written and oral form. Confidentiality of personal data and anonymity of subjects were protected. For this purpose, all data were coded and only researchers were allowed to access them.

## Results

Table 1 shows means, SD, and matrix of correlations of the study variables:

**TABLE 1:** Means, standard deviations, and correlations of variables in the model.

	M	SD	1	2	3	4	5	6
1. Effort	11.17	3.72						
2. Reward	22.40	7.88	0.66**					
3. Emotional exhaustion	12.83	10.34	0.61**	0.46**				
4. Gender (woman = 2)	1.84	0.36	0.13	0.13	0.09*			
5. Age (years)	42.06	12.41	–0.10	–0.13	−0.19**	0.11**		
6. Experience (years)	12.37	11.18	–0.11	−0.18*	−0.13**	0.03	0.77**	
7. Contract (permanent = 2)	1.81	0.39	–0.12	–0.11	−0.10*	0.03	0.37	0.35**

*p < 0.05; **p < 0.01; ***p < 0.00.

From the results in Table 1 we observed the strong and significative correlations among the variables effort, reward and emotional exhaustion, although we were not able to observe correlation relationships with socio-demographical gender and age variables, with the exception of years of experience.

In addition, to check whether our data had a normal distribution, we applied two goodness-of-fit tests (Kolmogorov–Smirnov test and Shapiro–Wilk test) which obtained very good scores above 0.05 (K–S = 0.90 *p* = 0.00 and W = 0.11 *p* = 0.00, respectively).

**FIGURE 1:**
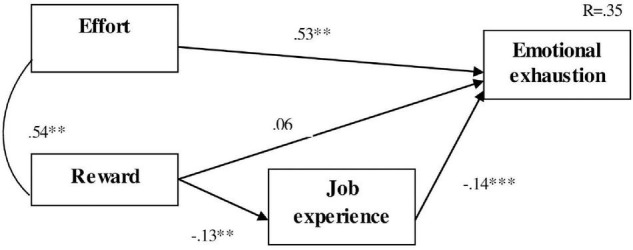
Effort-reward model. **p < 0.01; ***p < 0.001.

In order to analyze the relationships between the different study variables, we designed a model (see Figure 1), which was analyzed using structural equation modeling (SEM) techniques. Specifically, we analyzed the effects of the two independent variables observed (Effort and Reward) on the dependent variable “Emotional Exhaustion” and the influence of the organizational variable “Job experience” as a mediating variable. In addition, standardized coefficients and the coefficient of determination (R2) were included in the model.

Figure 1 shows that the variable “Effort” is the one that presents the strongest and the most statistically significant direct effect on “Emotional Exhaustion” (β = 0.53, *p* < 000), which would support our Hypothesis 1. However, the variable “Reward” did not have a statistically significant direct effect on “Emotional Exhaustion,” therefore we could not support our Hypothesis 2.

Regarding the Hypothesis 3 in which we pointed out the mediating effect of the years of experience in these variables, we could only partially prove the data obtained (Hypothesis 3b). After observing the results, we can appreciate that the effect the variable “Reward” produces on the dependent variable may be indirect and, in that case, be mediated by “Job experience.” All these variables manage to explain 35% of “Emotional Exhaustion” variability.

However, fit in SEMs is determined by the indexes that express the degree of concordance between the data predicted by the model and the observed data. Fit indexes help measure whether the model fits well enough to provide a useful approximation of reality and a reasonable explanation of data trends. In our case, Table 2 contains the main goodness of fit indexes for the evaluation of our model.

**TABLE 2:** Fit index of the effort-reward model.

χ ^2^	CMIN/DF	GFI	AGFI	NFI	CFI	RMSEA	PRATIO
0.855 *df* = 1	0.85	0.99	0.99	0.99	1.00	0.00	0.17

The value of χ^2^ was not significant (*p* = 0.26). This figure shows a good fit and allows us to accept the model; however, given the instability that χ^2^, we complement this information with other goodness of fit indexes such as the CMIN/DF index, which is less sensitive to sample size issues. The value of this index is below the levels mentioned by the authors, which reinforces the idea that the model is acceptable. GFI and AGFI are close to 1, which demonstrates an excellent overall fit of the model. Moreover, the incremental fit indicators NFI and CFI present a very good fit, coming close to unity. The RMSEA (0.00) is well below the threshold established by the authors, which provides evidence in favor of the acceptance of the model. To sum up, the proposed model obtained good fit indexes.

## Discussion

Our study allowed us to have a more comprehensive view of the importance of the ERI model in the burnout processes, especially in the variable “Reward” and how this relation is measured by other occupational variables. Yet, although the importance of both variables has been proved, we have also looked into the relationship that job experience has on professional burnout.

The proposed model, as well as the analysis of goodness and relationship between variables, showed that burnout is affected by their overstrain and by the low rewards perceived from their performance, although in the latter case, this relationship is mediated by work experience.

Thus, in general terms, we can affirm that job experience plays an important role in workers’ chronic stress. These results would be in line with the findings found by other authors (Brewer and Shapard, 2004; Bretones and González, 2011; Kruczek et al., 2020) who showed that occupational factors had a predictive role in employees’ exhaustion.

### Practical Implications

The findings of our study recover the value of job experience as a personal resource that shows a mediating effect on the perception of the reward received from their performance and its effect on professional burnout.

Thus, employees with greater job experience will better deal with situations of emotional distress as a reaction against the lack of reciprocity between overstrain and the benefits obtained from that performance (Feuerhahn et al., 2012) because they have developed better coping skills and with them they can minimize the level of professional burnout and increase subjective wellbeing (Leonova et al., 2019).

From these results, we could confirm that high effort and low rewards are associated with professional burnout; in the first case directly and positively although in the second case, mediated by job experience, indirectly and negatively. Therefore, it is necessary for researchers to show greater interest in the study of mediating factors in the relationship of the effort-reward model and burnout, in order to specify the evaluation processes as well as to suggest proposals for the vocational training curriculum and the intervention strategies. All these studies will enable us to create healthier working environments (Jáimez and Bretones, 2011) as well as the development of their workers and the prevention of psychosocial risks at work.

### Limitations and Recommendations of the Study

The present study presents some limitations to taken into account for future research. First, the cross-sectional design of the study. Besides, we believe that it would be advisable to conduct new studies as well as in other samples and countries about the work factors that mediate in the relationship between the effort-reward model and burnout, in order to specify the evaluation processes as well as the intervention strategies. Our study has focused on nursing professionals, so it would be advisable to carry out research in other positions (health and non-health) in order to confirm our model. It would also be recommendable to perform studies in other countries and cultural contexts that would allow us to generalize the results found.

## Data Availability Statement

The raw data supporting the conclusions of this article will be made available by the authors, without undue reservation.

## Author Contributions

LA, JR, and FB: conceptualization and writing draft preparation. FB and JR: formal analysis and data curation. LA: project administration. All authors have read and agreed to the published version of the manuscript.

## Conflict of Interest

The authors declare that the research was conducted in the absence of any commercial or financial relationships that could be construed as a potential conflict of interest.

## Publisher’s Note

All claims expressed in this article are solely those of the authors and do not necessarily represent those of their affiliated organizations, or those of the publisher, the editors and the reviewers. Any product that may be evaluated in this article, or claim that may be made by its manufacturer, is not guaranteed or endorsed by the publisher.

## References

[B1] Almodibeg B. A. Smith H. (2021). A cross-sectional survey to explore the prevalence and causes of occupational burnout syndrome among perioperative nurses in Saudi Arabia. *Nurs. Open* 8 364–371. 10.1002/nop2.637 33318844 PMC7729769

[B2] Alvarado L. E. Bretones F. D. (2018). New working conditions and well-being of elementary teachers in Ecuador. *Teach. Teach. Educ.* 69 234–242. 10.1016/j.tate.2017.10.015

[B3] Backhaus O. Hampel P. Dadaczynski K. (2018). Effort-reward imbalance and burnout in German kindergarten educators. *Eur. J. Health Psychol.* 25 73–82. 10.1027/2512-8442/a000012

[B4] Bakker A. B. Kilmer C. Siegrist J. Schaufeli W. (2000). Effort ± reward imbalance and burnout among nurses. *J. Adv. Nurs.* 31 884–891. 10759985 10.1046/j.1365-2648.2000.01361.x

[B5] Bang H. Reio T. G. Jr. (2017). Examining the role of cynicism in the relationships between burnout and employee behavior. *Rev. Psicol. Trabajo Organ.* 33 217–227. 10.1016/j.rpto.2017.07.002

[B6] Barrett P. (2007). Structural equation modelling: adjudging model fit. *Pers. Individ. Dif.* 42 815–824. 10.1016/j.paid.2006.09.018

[B7] Basińska B. A. Wilczek-Rużyczka E. (2013). The role of rewards and demands in burnout among surgical nurses. *Int. J. Occup. Med. Environ. Health* 26 593–604. 10.2478/s13382-013-0129-8 24057207

[B8] Boateng Y. A. Osei S. A. Aboh I. K. Druye A. A. (2021). Causes of burnout syndrome and coping strategies among high dependency unit nurses of an institution in the greater Accra region of Ghana. *Nurs. Open*. 10.1002/nop2.1052 Online ahead of print 34468085 PMC8510731

[B9] Bretones F. D. González M. J. (2011). Subjective and occupational well-being in a sample of Mexican workers. *Soc. Indic. Res.* 100 273–285. 10.1007/s11205-010-9616-5

[B10] Brewer E. W. Shapard L. (2004). Employee burnout: a meta-analysis of the relationship between age or years of experience. *Hum. Resour. Dev. Rev.* 3 102–123. 10.1177/1534484304263335

[B11] Browne M. W. Cudeck R. (1993). “Alternative ways of assessing model fit,” in *Testing Structural Equation Models*, eds Bollen K. Long L. (Newbury Park, CA: Sage), 136–162.

[B12] Cañadas-de la Fuente G. A. San Luis C. Lozano L. M. Vargas C. García I. Emilia I. (2014). Evidencia de validez factorial del Maslach burnout inventory y estudio de los niveles de burnout en profesionales sanitarios. *Rev. Latinoam. Psicol.* 46 44–52. 10.1016/S0120-0534(14)70005-6

[B13] Chen F. Curran P. J. Bollen K. A. Kirby J. Paxton P. (2008). An empirical evaluation of the use of fixed cutoff points in RMSEA test statistic in structural equation models. *Sociol. Methods Res.* 36 462–494. 10.1177/0049124108314720 19756246 PMC2743032

[B14] Dall’Ora C. Ball J. Reinius M. Griffiths P. (2020). Burnout in nursing: a theoretical review. *Hum. Resour. Health* 18:41. 10.1186/s12960-020-00469-9 32503559 PMC7273381

[B15] Devonish D. (2018). Effort-reward imbalance at work: the role of job satisfaction. *Pers. Rev.* 47 319–333. 10.1108/PR-08-2016-0218

[B16] Diekmann K. Böckelmann I. Karlsen H. R. Lux A. Thielmann B. (2020). Effort-reward imbalance, mental health and burnout in occupational groups that face mental stress. *J. Occup. Environ. Med.* 62 847–852. 10.1097/JOM.0000000000001978 32769796

[B17] Ding Y. Qu J. Yu X. Wang S. (2014). The mediating effects of burnout on the relationship between anxiety symptoms and occupational stress among community healthcare workers in China: a cross-sectional study. *PLoS One* 9:e107130. 10.1371/journal.pone.0107130 25211025 PMC4161428

[B18] Dragano N. Siegrist J. Nyberg S. T. Lunau T. Fransson E. I Alfredsson L. (2017). Effort–reward imbalance at work and incident coronary heart disease. *Epidemiology* 28 619–626. 10.1097/EDE.0000000000000666 28570388 PMC5457838

[B19] Eddy P. Wertheim E. H. Hale M. W. Wright B. J. (2018). A systematic review and meta-analysis of the effort-reward imbalance model of workplace stress and hypothalamic-pituitary-adrenal axis measures of stress. *Psychosom. Med.* 80 103–113. 10.1097/PSY.0000000000000505 28731983

[B20] Feuerhahn N. Kühnel J. Kudielka B. M. (2012). Interaction effects of effort–reward imbalance and overcommitment on emotional exhaustion and job performance. *Int. J. Stress Manag.* 19 105–131. 10.1037/a0028338

[B21] Garcia H. A. McGeary C. A. Finley E. P. Ketchum N. S. McGeary D. D. Peterson A. L. (2015). Burnout among psychiatrists in the Veterans Health Administration. *Burn. Res.* 2 108–114. 10.1016/j.burn.2015.10.001

[B22] Gilbert-Ouimet M. Trudel X. Brisson C. Milot A. Vézina M. (2014). Adverse effects of psychosocial work factors on blood pressure: systematic review of studies on demand-control-support and effort-reward imbalance models. *Scand. J. Work Environ. Health* 40 109–132. 10.5271/sjweh.3390 24108310

[B23] Giorgi G. Arcangeli G. Perminiene M. Lorini C. Ariza-Montes A. Fiz-Perez J. (2017). Work-related stress in the banking sector: a review of incidence, correlated factors, and major consequences. *Front. Psychol.* 8:2166. 10.3389/fpsyg.2020.623587 29312044 PMC5733012

[B24] Gorgievski M. J. Van der Heijden B. I. J. M. Bakker A. B. (2019). Effort-reward imbalance and work-home interference: a two-wave study among European male nurses. *Work Stress* 33 315–333. 10.1080/02678373.2018.1503358

[B25] Guerrero-Barona E. Guerrero-Molina M. García-Gómez A. Moreno-Manso J. M. García-Baamonde M. E. (2020). Quality of working life, psychosocial factors, burnout syndrome and emotional intelligence. *Int. J. Environ. Res. Public Health* 17:9550. 10.3390/ijerph17249550 33419344 PMC7767310

[B26] Hair J. F. Black W. C. Babin B. J. Anderson R. E. (2009). *Multivariate Data Analysis.* New York, NY: Prentice Hall.

[B27] Hanson E. K. Schaufeli W. Vrijkotte T. Plomp N. H. Godaert G. L. (2000). The validity and reliability of the dutch effort-reward imbalance questionnaire. *J. Occup. Health Psychol.* 5 142–155. 10.1037/1076-8998.5.1.142 10658892

[B28] Hwang J. E. Kim N. J. Kwon N. Kim S. Y. (2019). An effort-reward imbalance model to study engagement and burnout: a pilot study. *J. Educ. Dev.* 3:1.

[B29] Iacobucci D. (2008). “Mediation analysis,” in *Quantitative Applications in the Social Sciences*, ed. Lia T. F. (Thousand Oaks, CL: Sage), 1–85.

[B30] Jachens L. Houdmont J. Thomas R. (2019). Effort–reward imbalance and burnout among humanitarian aid workers. *Disasters* 43 67–87. 10.1111/disa.12288 29893486

[B31] Jáimez M. J. Bretones F. D. (2011). Towards a healthy organisation model: the relevance of empowerment. *ISGUC J. Indust. Relat. Hum. Resour.* 13 7–26. 10.4026/1303-2860.2011.0180.x

[B32] Juárez A. Vera-Calzaretta A. Blanco-Gomez G. Gómez-Ortiz V. Hernández-Mendoza E. Jacinto-Ubillus J. (2015). Validity of the effort / reward imbalance questionnaire in health professionals from six Latin-American countries. *Am. J. Indust. Med.* 58 636–649. 10.1002/ajim.22432 25919593

[B33] Juvani A. Oksanen T. Virtanen M. Salo P. Pentti J. Kivimäki M. (2018). Clustering of job strain, effort- reward imbalance, and organizational injustice and the risk of work disability: a cohort study. *Scand. J. Work Environ. Health* 44 485–495. 10.5271/sjweh.3736 29777612

[B34] Koutsimani P. Anthony M. Georganta K. (2019). The relationship between burnout, depression and anxiety: a systematic review and meta-analysis. *Front. Psychol.* 10:284. 10.3389/fpsyg.2019.00284 30918490 PMC6424886

[B35] Kruczek A. Basińska M. A. Janicka M. (2020). Cognitive flexibility and flexibility in coping in nurses–the moderating role of age, seniority and the sense of stress. *Int. J. Occup. Med. Environ. Health* 33 507–521. 10.13075/ijomeh.1896.01567 32541970

[B36] Leineweber C. Bernhard-Oettel C. Eib C. Peristera P. Li J. (2021). The mediating effect of exhaustion in the relationship between effort-reward imbalance and turnover intentions: a 4-year longitudinal study from Sweden. *J. Occup. Health* 63:e12203. 10.1002/1348-9585.12203 33543549 PMC7862986

[B37] Leka S. Jain A. Lerouge L. (2017). “Work-related psychosocial risks: key definitions and an overview of the policy context in Europe,” in *Psychosocial Risks in Labour and Social Security Law*, ed. Lerouge L. (Cham: Springer), 1–12. 10.1007/978-3-319-63065-6_1

[B38] Leonova I. S. Zakharova L. N. Bretones F. D. (2019). Subjective well-being of russian female personnel as an indicator of socio-psychological age. *Opción* 35 962–982.

[B39] Leon-Perez J. M. Antino M. Leon-Rubio J. M. (2016). The role of psychological capital and intragroup conflict on employees’ burnout and quality of service: a multilevel approach. *Front. Psychol.* 7:1755. 10.3389/fpsyg.2016.01755 27895601 PMC5107570

[B40] Makara-Studzińska M. Wajda Z. Lizińczyk S. (2020). Years of service, self-efficacy, stress and burnout among Polish firefighters. *Int. J. Occup. Med. Environ. Health*. 33 283–297. 10.13075/ijomeh.1896.01483 32210420

[B41] Maslach C. Jackson S. E. (1981). The measurement of experienced burnout. *J. Organ. Behav.* 2 99–113. 10.1002/job.4030020205

[B42] Maslach C. Schaufeli W. B. Leiter M. P. (2001). Job burnout. *Annu. Rev. Psychol.* 52 397–422. 10.1146/annurev.psych.52.1.397 11148311

[B43] Olivares-Faúndez V. E. Mena-Miranda L. Jélvez-Wilke C. Macía-Sepúlveda F. (2014). Validez factorial del Maslach burnout inventory human services (MBI-HSS) en profesionales Chilenos. *Univ. Psychol.* 13 145–160. 10.11144/Javeriana.UPSY13-1.vfmb

[B44] Oren L. Littman-Ovadia H. (2013). Does equity sensitivity moderate the relationship between effort-reward imbalance and burnout. *Anxiety Stress Coping* 26 643–658. 10.1080/10615806.2012.753060 23286362

[B45] Ortega-Jiménez D. Ruisoto P. Bretones F. D. Ramiírez M. R. Vaca Gallegos S. (2021). Psychological (In)flexibility mediates the effect of loneliness on psychological stress. evidence from a large sample of university professors. *Int. J. Environ. Res. Public Health* 18:2992. 10.3390/ijerph18062992 33803920 PMC8001878

[B46] Peiró J. M. González-Romá V. Tordera N. Mañas M. A. (2001). Does role stress predict burnout over time among health care professionals? *Psychol. Health* 16 511–525. 10.1080/08870440108405524 22804496

[B47] Portoghese I. Galletta M. Coppola R. C. Finco G. Campagna M. (2014). Burnout and workload among health care workers: the moderating role of job control. *Saf. Health Work* 5 152–157. 10.1016/j.shaw.2014.05.004 25379330 PMC4213899

[B48] Rantanen J. Feldt T. Hyvönen K. Kinnunen U. Mäkikangas A. (2013). Factorial validity of the effort-reward imbalance scale: evidence from multi-sample and three-wave follow-up studies. *Int. Arch. Occup. Environ. Health* 86 645–656. 10.1007/s00420-012-0798-9 22824906

[B49] Rugulies R. Aust B. Madsen I. E. (2017). Effort–reward imbalance at work and risk of depressive disorders. A systematic review and meta-analysis of prospective cohort studies. *Scand. J. Work Environ. Health* 43 294–306. 10.5271/sjweh.3632 28306759

[B50] Salami S. O. Ajitoni S. O. (2016). Job characteristics and burnout: the moderating roles of emotional intelligence, motivation and pay among bank employees. *Int. J. Psychol.* 51 375–382. 10.1002/ijop.12180 26118824

[B51] Salvagioni D. A. J. Melanda F. N. Mesas A. E. González A. D. Gabani F. L. Andrade S. M. D. (2017). Physical, psychological and occupational consequences of job burnout: a systematic review of prospective studies. *PLoS One* 12:e0185781. 10.1371/journal.pone.0185781 PMC5627926 28977041

[B52] Siegrist J. (1996). Adverse health effects of high-effort / low-reward conditions. *J. Occup. Health Psychol.* 1 27–41. 10.1037/1076-8998.1.1.27 9547031

[B53] Siegrist J. Li J. (2016). Associations of extrinsic and intrinsic components of work stress with health: a systematic review of evidence on the effort-reward imbalance model. *Int. J. Environ. Res. Public Health* 13:432. 10.3390/ijerph13040432 27104548 PMC4847094

[B54] Siegrist J. Dragano N. Nyberg S. T. Lunau T. Alfredsson L. Erbel R. (2014). Validating abbreviated measures of effort-reward imbalance at work in European cohort studies: the IPD-Work consortium. *Int. Arch. Occup. Environ. Health* 87 249–256. 10.1007/s00420-013-0855-z 23456220

[B55] Siegrist J. Starke D. Chandola T. Godin I. Marmot M. Niedhammer I. (2004). The measurement of effort-reward imbalance at work: European comparisons. *Soc. Sci. Med.* 58 1483–1499. 10.1016/S0277-9536(03)00351-4 14759692

[B56] Tian M. Yang H. Yin X. Wu Y. Zhang G. Lv C. (2021). Evaluating effort-reward imbalance among nurses in emergency departments: a cross-sectional study in China. *BMC Psychiatry* 21:353. 10.1186/s12888-021-03344-6 34261458 PMC8278678

[B57] Topa G. Guglielmi D. Depolo M. (2016). Effort-reward imbalance and organisational injustice among aged nurses: a moderated mediation model. *J. Nurs. Manag.* 24 834–842. 10.1111/jonm.12394 27169619

[B58] Van Mol M. M. C. Kompanje E. J. O. Benoit D. D. Bakker J. Nijkamp M. D. Seedat S. (2015). The prevalence of compassion fatigue and burnout among healthcare professionals in intensive care units: a systematic review. *PLoS One* 10:e0136955. 10.1371/journal.pone.0136955 26322644 PMC4554995

[B59] Wheeler D. L. Vassar M. Worley J. A. Barnes L. L. B. (2011). A reliability generalization meta-analysis of coefficient alpha for the Maslach burnout inventory. *Educ. Psychol. Meas.* 71 231–244. 10.1177/0013164410391579

[B60] Xie Z. Wang A. Chen B. (2011). Nurse burnout and its association with occupational stress in a cross-sectional study in Shanghai. *J. Adv. Nurs.* 67 1537–1546. 10.1111/j.1365-2648.2010.05576.x 21261698

